# Genome-wide discovery of missing genes in biological pathways of prokaryotes

**DOI:** 10.1186/1471-2105-12-S1-S1

**Published:** 2011-02-15

**Authors:** Yong Chen, Fenglou Mao, Guojun Li, Ying Xu

**Affiliations:** 1 Computational Systems Biology Laboratory, Department of Biochemistry and Molecular Biology, Institute of Bioinformatics, University of Georgia, Athens, GA 30602, USA; 2 BioEnergy Science Center (http://bioenergycenter.org/), USA; 3 School of Mathematics, Shandong University, Jinan, Shandong 250100, China; 4 School of Sciences, University of Jinan, Jinan, Shandong 250022, China; 5 MOE Key Laboratory of Bioinformatics and Bioinformatics Division, TNLIST/Department of Automation, Tsinghua University, Beijing 100084, China; 6 College of Computer Science and Technology, Jilin University, Changchun, China

## Abstract

**Abstract:**

**Background**

Reconstruction of biological pathways is typically done through mapping well-characterized pathways of model organisms to a target genome, through orthologous gene mapping. A limitation of such pathway-mapping approaches is that the mapped pathway models are constrained by the composition of the template pathways, e.g., some genes in a target pathway may not have corresponding genes in the template pathways, the so-called “missing gene” problem.

**Methods**

We present a novel pathway-expansion method for identifying additional genes that are possibly involved in a target pathway after pathway mapping, to fill holes caused by missing genes as well as to expand the mapped pathway model. The basic idea of the algorithm is to identify genes in the target genome whose homologous genes share common operons with homologs of any mapped pathway genes in some reference genome, and to add such genes to the target pathway if their functions are consistent with the cellular function of the target pathway.

**Results**

We have implemented this idea using a graph-theoretic approach and demonstrated the effectiveness of the algorithm on known pathways of *E. coli* in the KEGG database. On all KEGG pathways containing at least 5 genes, our method achieves an average of 60% positive predictive value (PPV) and the performance is increased with more seed genes added. Analysis shows that our method is highly robust.

**Conclusions**

An effective method is presented to find missing genes in biological pathways of prokaryotes, which achieves high prediction reliability on *E. coli* at a genome level. Numerous missing genes are found to be related to knwon *E. coli* pathways, which can be further validated through biological experiments. Overall this method is robust and can be used for functional inference.

## Background

Reconstruction of biological pathways is a fundamental problem in understanding the functional mechanisms of cellular organisms. Substantial efforts have been put into the elucidation of biological pathways, particularly for prokaryotic organisms, in a systematic manner based on high-throughput *omic* data and computational prediction. As a result, a number of pathway databases have been developed and are being widely used, such as KEGG and BioCyc [1-5]. These databases not only serve as an information resource for retrieving well-characterized pathways for specific organisms but also provide a set of pathway templates for reconstructing pathways for organisms that are not directly covered by the databases, as substantial portions of homologous pathways may be conserved across different organisms, particularly related organisms.

A number of computer programs have been developed for pathway reconstruction through mapping known pathways from one organism to another. While some success has been reported on these programs, there has been a general issue associated with such homologous pathway mapping-based approaches, which is that homologous pathways are generally not identical and hence the mapped pathways could miss some parts not covered by their well-characterized homologous template pathways. This problem, called *pathway holes* or *missing genes*, has been widely recognized [6-9]. A number of methods have been developed to find such missing genes, based mainly on the idea of finding genes that are functionally associated with genes already in the mapped pathways. One class of such methods attempts to find enzyme-encoding genes missing in a mapped metabolic pathway based on multiple types of gene association information [8-10], taking advantage of the fact that genes encoding a metabolic pathway tend to group into clusters (e.g., operons). Another class of methods attempt to identify functional modules from some large gene association networks or groups [11-15], and then to suggest possible candidates for missing genes based on genes found in the same functional modules of genes already in mapped pathways. While these methods have provided useful information for searching for missing genes, there is clearly substantial room for improvement in terms of the functional specificity of their predicted candidates and the scope of applicability of the existing methods [16]. Among the various areas for further improvements, we identified a few we can possibly improve on using the currently available information: (i) there have not been reliable methods for consideration and inclusion of functionally uncharacterized genes (often referred to as *hypothetical* and *conserved genes*) into partially predicted pathway models (e.g, mapped pathways); (ii) while (conserved) genomic synteny has been utilized for prediction of functionally associated genes, its true usefulness, other than operon information, is yet to be well documented. Previous studies have shown that there is a strong link between genes in the same operons and genes working in the same biological pathways [17]. So full utilization of operon information should be a key direction for improving biological pathways, particular now as the state of the art prediction methods for operons have reached high accuracy (~90%) [17-19].

We present, in this paper, a novel computational method for identification and functional annotation of missing genes in a predicted pathway model, either through homologous pathway mapping or using other methods. The basic idea of the method can be outlined as follows. For any specified target genome, we define a distance between any pair of genes in the genome to measure the level of their functional relatedness in terms of a set of reference genomes. Specifically, two genes are functionally *related* if they (i) are homologous, (ii) share a common operon directly or through their homologs in a reference genome, (iii) are phylogenetically related, or (iv) deemed to be functionally related through combinations of the first three criteria. For any pair of functionally related genes in the target genome, their distance is defined essentially as the minimum number of applications of this recursive definition. Our algorithm identifies genes possibly involved in a target pathway based on their distances to genes already in the pathway. We have tested the algorithm on all characterized pathways of *E. coli*, using portions of the pathways as the initial pathway genes (called *seed*s), and found that the vast majority of the remaining genes of these known pathways are all within short distances to the seeds, confirming the effectiveness of our distance measure. Our study has also identified numerous genes with short distances to the known pathway genes, which we believe are highly promising candidates for addition to these known *E. coli* pathways. Limited analyses of the potential functional roles of these genes have been carried out, and reported in this paper.

## Methods

### High-level description of our algorithm

**Figure 1:**
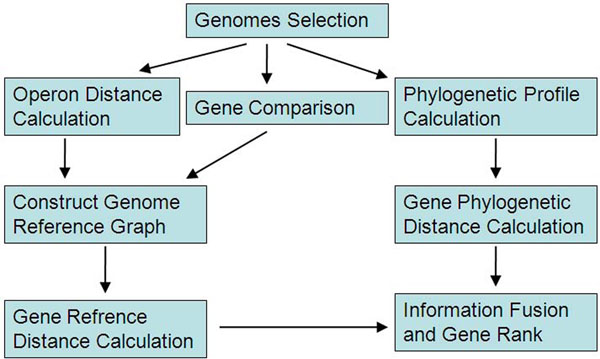
**The flow chart of the method.** The method uses gene similarity and operon information to first construct a genome reference graph. It then hierarchically fuses the shortest path distance and phylogenetic distance to rank all candidate genes.

We first represent genes in the target genome or in a set of specified reference genomes, and their functional relatedness as a graph, called a *reference graph*, where each gene in any of the genomes is represented as a vertex, and two genes have an edge linking them if they are in the same operon or they are homologous. We then define a *linkage graph* for the target genome such that each gene is represented as a vertex and two genes have an edge if and only if there is a path linking the two genes in the reference graph, and the distance of the edge is defined as the distance of the shortest path between the two genes in the reference graph. We have augmented this distance by including two additive terms, one penalty factor (*system*(*error*)) used to model the reliability of a predicted functional relationship, and a *phylogeny-based distance* used to capture co-evolutionary relationships, more general than homology relationships among genes, between two genes. Our goal here is to find genes that have short distances, defined above, to genes in a known pathway, and predict that they are involved in this pathway if their distances are ranked among the top such genes. The whole procedure is summarized in Figure 1, with the detailed steps explained as follows**.**

### Selection of reference genomes

Currently over 1,000 bacterial and archaean genomes have been sequenced and are publicly available (NCBI release of September 2009). From this set, we have selected 185 strains (non-redundant genomes and plasmids) (see Additional File 1) from 185 different genera using the following rule: for each genus, select the genome with the longest sequence.

### Calculation of homology-based distance

For each pair of genes *x_i_*, *x_j_*, in the target genome and the 185 reference genomes, we use the E-value of BLAST (with default parameters) to define their *homology-based distance d_s_*(*x_i_*, *x_j_*) as follows:(1)

where *p_s_*(*x_i_*, *x_j_*) is the BLAST E-value for genes *x_i_*, *x_j_*, and 185 is a normalization factor since when the E-value is smaller than 1e-185, it is set as 0 in the BLAST program. Clearly *d_s_*(*x_i_*, *x_j_*) is between 0 and 1; and the more similar two genes are, the smaller the *d_s_*(*x_i_*, *x_j_*) value is.

### Calculation of operon-based distance

We have used the operons predicted using our own program [18], which is considered the most reliable operon prediction method in the public domain [17]. A probability calculated by this method represents the likelihood that two neighbouring genes are in the same operon. We apply this program to all of the 185 reference genomes and get the probability *p_o_*(*x_i_*, *x_j_*) between two genes *x_i_*, *x_j_* in each genome. For any pair of neighbouring genes *x_i_*, *x_j_* in the same genome (target or reference), we define their *operon-based distanced_o_*(*x_i_*, *x_j_*) as follows:(2)

where *p_o_*(*x_i_*, *x_j_*) represents the probability that *x_i_*, *x_j_* are in the same operon as given in [18].

### Reference graph and linkage graph

**Figure 2:**
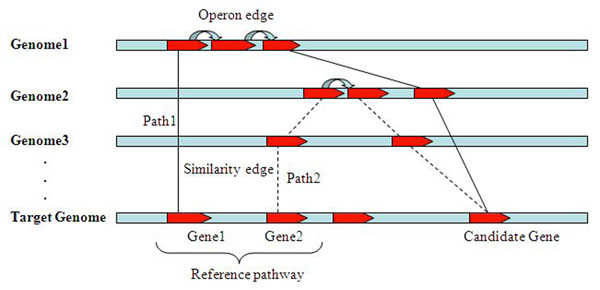
**The relationship path through operon edge and similarity edge.** Given a reference pathway, its known genes are used as seeds to calculate the shortest distances to candidate genes. For example, gene1 and gene2 are connected with the same candidate gene. The path from gene1 to candidate gene (path1) is noted as solid line, and gene2 to candidate gene (path2) as dashed line. The paths are both constructed by operon edge (colour arrow) and similarity edge (solid or dashed line).

We define a *reference graph* over all genes in the target as well as the reference genomes as follows. Each gene is represented as a vertex, and an edge between two genes is created if (i) the two genes are in the same operon, with their edge distance defined to be the operon-based distance between the two genes; or (ii) the two genes in different genomes are homologous, with their edge distance defined to be their homology-based distance. Based on the reference graph, we define a *linkage graph* on genes in the target genome. For any pair of genes, *x_i_*, *x_j_*, we define an edge between them if and only if there is a path *x_i_*, *x*_1_,*x*_2_, … *x_j_* in the reference graph, with its edge distance set to be the distance of the shortest path between the two genes (Figure 2). We intend to use an edge in this graph to capture a functional linkage relationship possibly through multiple steps of co-operon and homologous relationship. We recognize that the reliability of such defined edges could go down (largely independent of the reliability of individual operon and homology predictions) as the number of edges in the above path goes up. Hence we included a penalty factor, *system* (*error*), which is proportional to the number of edges in the path, and redefined the path distance of a gene pair as follows:(3)

where *k* is the number of edges in the path, and *α* is a scaling factor. In our current implementation, we set *α* = 380 and *system*(*error*) = 0.06 based on a ten-fold cross-validation method (see Parameter Selection). *E*(*operon*) and *E*(*similarity*) are the set of operon edges and the set of similarity edges, respectively.

### Phylogeny-based distance

We also considered a more general class of functional relationship defined in terms of the phylogenetic profiles of genes, which measures their co-evolutionary relationship [20,21]. Basically, the phylogenetic profile *X* of a gene against a set of *n* reference genomes is a binary string of length *n*, with the *i*th position being 1, if the gene has a homolog in the *i*th reference genome, and 0 otherwise. It has been found that two genes (of the same genome) are generally functionally related if their phylogenetic profiles are highly similar [20]. We have used a BLAST E-value e-3 as the cutoff for determining the presence of a homolog in another genome [22]. We use the following to measure the similarity between two phylogenetic profiles, similar to that reported in [23]. Given the phylogenetic profiles *X*_i_ and *Y_j_* for genes *x_i_* and *y_j_*, their *phylogeny-based distance* is defined as follows:(4)

where, *d_hamming_*(*X_i_*, *X_j_*) is the Hamming distance between *X_i_* and *X_j_*, and *Entropy* (*X_i_*, *X_j_*) is the entropy of the common part of *X_i_* and *X_j_*, defined as follows:(5)

with *p* being the frequency of 1’s in common positions between the two phylogenetic profiles. Note that the more similar two phylogenetic profiles are, the smaller their distance is.

### Rank functional relatedness of candidate genes

Our goal here is to rank all the genes in a target genome in terms of a possible relationship with a set of seed genes (known genes in a pathway), by fusing the path distance and the phylogeny-based distance. For a given pathway *P*, let its known gene set be *G*(*P*) and |*G*(*P*)| be the number of its genes. We define a distance from *P* to a candidate gene *x_i_* as(6)

Similarly, we define a phylogenetic distance from *P* to *x_i_*as(7)

Our experience has been that for both the path distance and the phylogenetic distance, the distance for the top ranked genes tend to be more reliable. Hence only the top K candidate genes to each gene *x_j_* ε *G*(*P*) are considered and the remaining is ignored. To a seed gene, we only take the K shortest genes measured by reference distance, where the K is ranged from 5 to 30. Similarly, only the top K( = 50) genes closed to a seed gene is considered for phylogenetic distance [20]. So some candidate genes may not have a path distance or phylogenetic distance, due to their ranking. The final combined distance from any gene *x_i_* to pathway *P* is defined as(8)

where *β* is a scaling factor and set to 5, based on the ten-fold cross-validation method (see Parameter Selection); and T is set to be 2 if gene *x_i_* has both the path distance and phylogenetic distance, and as 1 if it has only one distance defined. The candidate genes are ranked by their combined distance and the final top *γ* genes are output (*γ* = 10 in this study).

### Parameter Selection and Validation Method

For a predicted target gene and a target pathway, the gene is considered a *positive* prediction (based on a partial gene list of the pathway) if it is part of the pathway. For any of the following assessments of our prediction, we use the following (standard) notations: TP for true positive predictions; TN for true negative predictions; FP for false positive predictions and FN for false negative predictions (FN); and we use the following standard measures of sensitivity (SE), specificity (SP) and positive predictive value (PPV) to assess the performance of our prediction method of missing genes:(9)(10)(11)

To assess the prediction performance against a set of pathways, we use the average of the above three measures across all the pathways as follows:(12)(13)(14)

where *SP_i_*, *SE_i_* and *PPV_i_* are *SP*, *SE* and *PPV* for the *i*th pathway, respectively, and *N* is the number of pathways considered.

For each to-be-determined parameter in our program, a ten-fold cross-validation procedure is used to derive the optimal value. Specifically, all the pathways are divided randomly into ten parts, nine for training and one for testing each time. The value with the best average is finally selected. The leave-one-out cross-validation procedure is used to assess the performance. For each pathway, its known genes are used as the seed-gene set. The procedure removes each gene from the pathway seed set one at a time, and then calculates the final combined distance from the remaining genes to the removed gene and all the left genes of the target genome. If the removed gene is output in the final top *γ* genes, it is counted as a successful prediction.

## Results

### Performance measure calculation

**Figure 3:**
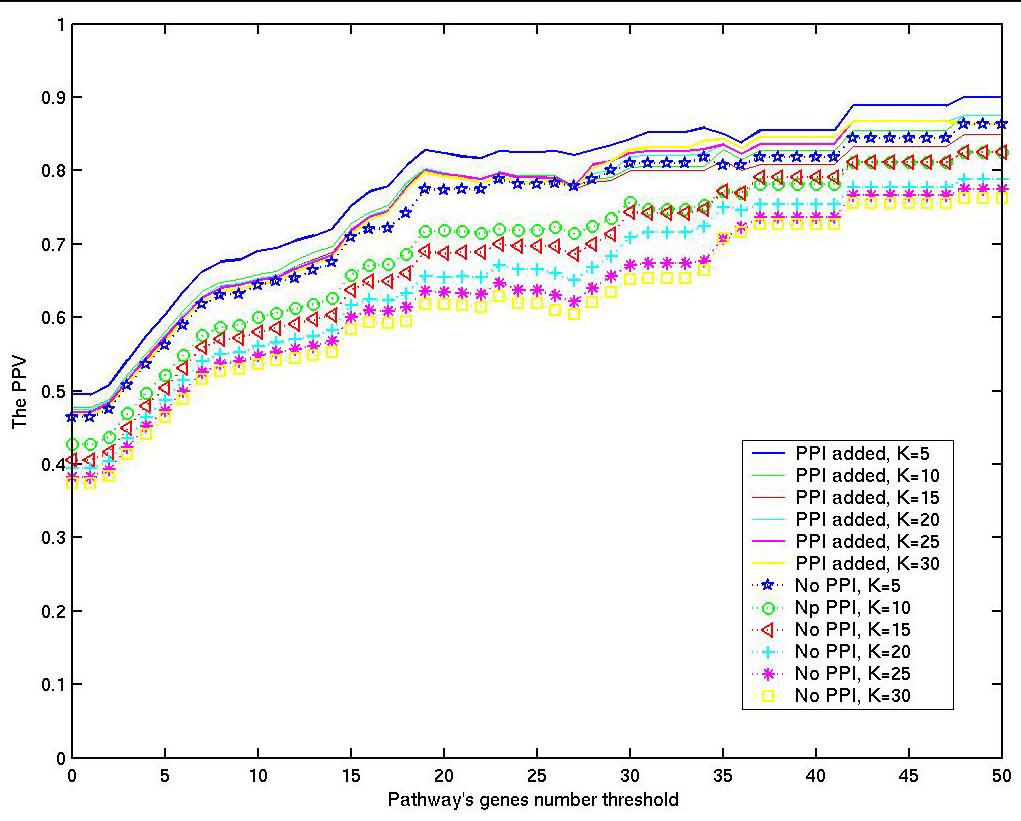
**The PPV rate based on a different number of pathway known genes.** The average PPV rate of pathways *P* with |*G*(*P*)| ≥ *x* is calculated, and *x* is the threshold number, changed from 1 to 50. *system*(*error*) = 0.06, *α* = 380, *β* = 5, *γ* = 380, K is changed to 5, 10, 15, 20, 25, 30. (PPI: Phylogenetic Profile Information)

We first tested our ranking algorithm on all the 121 KEGG pathways of *E. coli* K12. We have downloaded these pathways from KEGG (released in September of 2009; see Additional File 2), of which 105 are metabolic pathways, 11 are involved in genetic information processing, and 5 are involved in environmental information processing. On these 121 pathways, the performance of our method was tested with different K ranging from 5 to 30. Figure 3 shows the accuracies of our algorithm for different K and for pathways with different numbers of assigned genes. It has near 90% prediction accuracy (PPV) for K = 5, and the accuracy increases as the number of genes in a pathway increases. Also we noted that the PPV value decreases with the increase of the K value in general, suggesting a higher level of noise is being included as K increases. We have also calculated the SP and SE values for different K on 121 pathways; the detailed data is shown in Additional File 3. We noted that SE increases with the increase of K, achieving near 78% since only the top K shortest genes were considered.

While the major contribution to the prediction accuracy by our method is from operon and homology information, we have also assessed the contribution from phylogentic profiles. We noted that the phylogenetic profile gives a small increase for PPV (~ 4% for *K* = 5). When K increases, the contribution also increases (Figure 3). It shows that genes confirmed by the phylogenetic profile can reduce the mis-predicted genes from the graph-based prediction results, and increase the PPV value. This result suggests that phylogenetic profile can detect some genes which cannot be found by operon or sequence similarity alone.

One interesting observation we made is that our method gives rise to different performance levels for pathways in different functional categories. To fully investigate this observation, we have tested our algorithm on 18 different functional categories of KEGG pathways where each has at least 5 (assigned) genes. One special care needs to be taken when assessing the prediction performance as some KEGG pathways are predicted to form one “combined” pathway by our prediction. For example, all the pathways in Amino Acid Metabolism are put together into one combined “pathway”. Hence we need to evaluate the performance of our method on this combined “pathway”. The performance on the 18 categories of KEGG pathways is generally good except for the category of Biosynthesis of Secondary Metabolism, Metabolism of Other Amino Acids, Transcription and Xenobiotics Biodegradation and Metabolism (see Additional File 4). The reduced performance may be due to two reasons: (i) some correctly predicted genes are regarded as false positives since the combined pathway is incomplete; and (ii) the combined pathways may not be conserved across different genomes; and hence cannot be inferred by our method. We also calculated the PPV values of individual pathways whose number of genes is at least 30. They all have high prediction accuracy except for the Pyruvate Metabolism Pathway, which only gets 40% prediction accuracy (see Additional File 5).

### Case study of the predicted pyruvate metabolism pathway

We have carefully analyzed our prediction results on the pyruvate metabolism pathway (eco00620) since it has the worst prediction performance among all the 21 *E. coli* pathways, each of which has at least 30 (assigned) genes. This KEGG pathway currently consists of 41 annotated genes (released in September 2009); five (*pflD, tdcE, pflB, accC, ybiW*) of them are correctly predicted in the top 10 by our method. Among the “incorrect” top 10 predictions (*tdcD, eutD, ybiY, prpE, yiaY*), some have been reported as correct genes involved in the pathway by a number of published papers. For example, gene *ybiY* is predicted as a “pyruvate formate lyase activating enzyme” in the NCBI and KEGG databases. Furthermore, we find three genes (*tdcD, pflD, prpE*) are all in the same “Propanoate Metabolism” pathway (eco00640), which is directly related to the pyruvate metabolism pathway. Actually, there are 10 genes that are common in both pathways.

**Figure 4:**
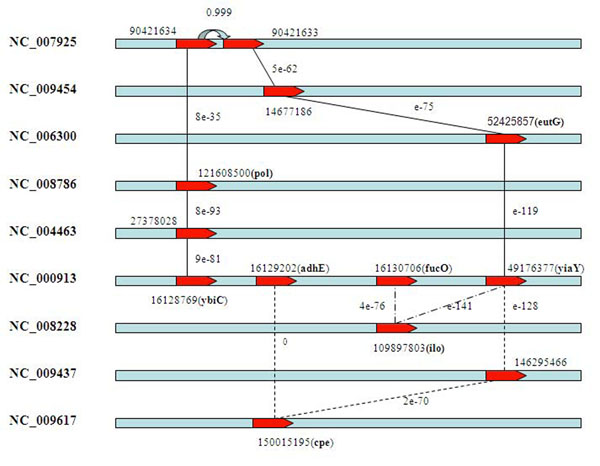
**The connected paths to candidate gene *yiaY.*** The three paths from *ybiC*, *adhE* and *fucO* to *yiaY* in the genome reference graph are presented and noted with the original operon probability and the BLAST similarity. The NCBI gene id is used to the connected genes and the gene symbol is noted in bracket.

**Figure 5:**
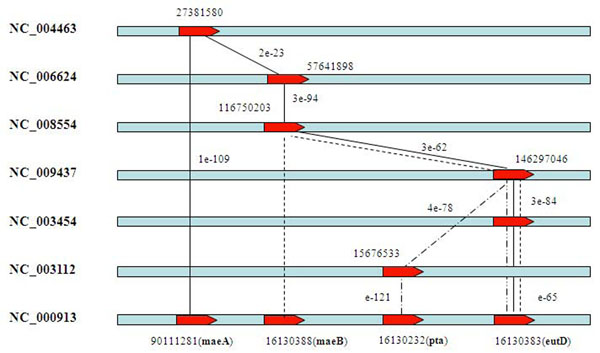
**The connected paths to candidate gene *eutD***. The three paths from *maeA*, *maeB* and *pta* to *eutD* in the genome reference graph are presented and noted by original BLAST similarity. The NCBI gene id is used to the connected genes and the gene symbol is noted in bracket.

Gene *yiaY* is annotated as “Fe-containing alcohol dehydregenas” and is predicted among the top 5 predictions by three known pathway genes (*ybiC, adhE, fucO*) with the similarity connection path or the operon connection path (Figure 4). Both gene *ybiC* and gene *yiaY* have homologous genes in genome (NC_007925) and are reported as an operon with high probability (≥ 0.999). The connections show that these two genes are structured as an operon in NC_007925, while they are diverged into different segments in *E. coli*. The gene *eutD* is ranked among the top 5 predictions by three pathway genes (*maeA*, *maeB* and *pta*) (Figure 5) connected by a similarity connection. These results suggested that our method can give a reasonable gene rank list to a target pathway.

### Robustness analysis of parameters

**Figure 6:**
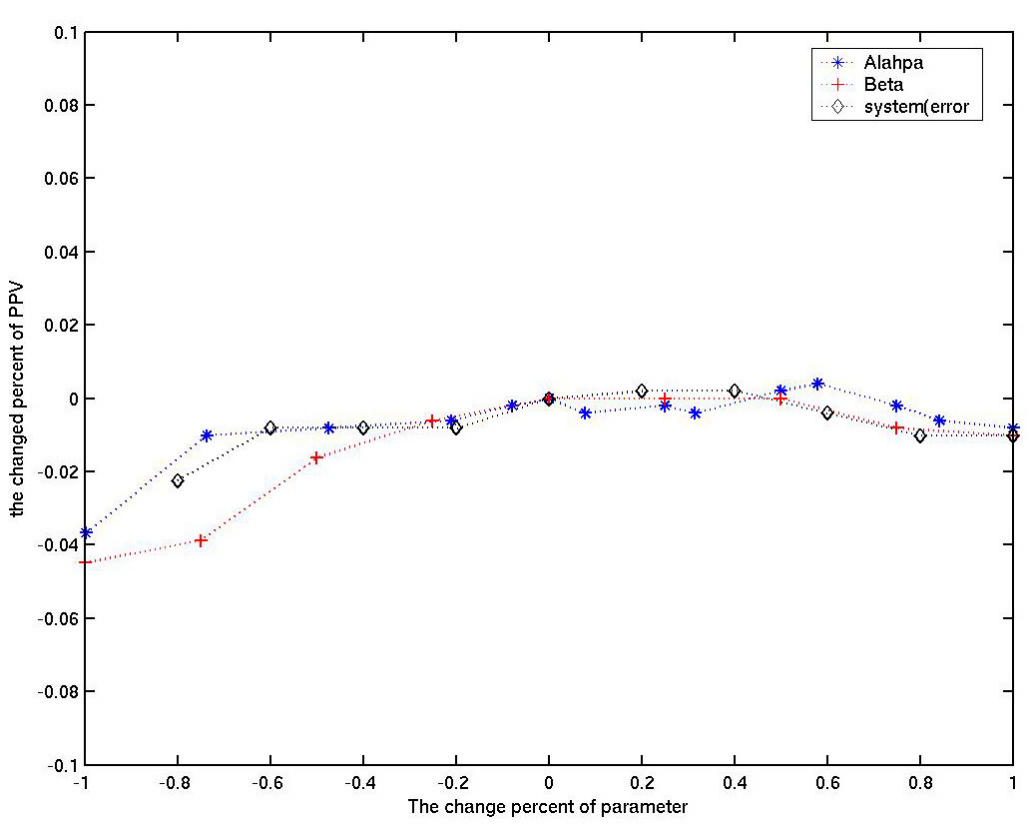
**The robustness of parameters *α*, *β* and *system*(*error*).** Three parameters (X-axis) are changed ±100% compared with the value used in our study and the final accuracy change rates are described in the Y-axis.

To test the robustness of our method, we calculated the change in the average PPV value when the parameters *α*, *β* and *system*(*error*) change. The initial parameter values are set as *K* = 5, *α* = 380 and *system*(*error*) = 0.06. 71 pathways (with the number of assigned genes ≥ 10) are used, and the final average PPV of the top 10 genes are calculated. For parameter *x*, the change rate is defined as  and the related PPV change rate is . For each parameter, the change rate ranges from -1 to 1. The results show that our method is very robust in terms of these three parameters. For example, when the change rate of *system*(*error*) is -1, the related PPV change rate is only 0.0449 (Figure 6). It is a very small change compared with the change of *system*(*error*). This result also shows that the *system*(*error*) can give an extra 0.0449 contribution to the final average PPV; and suggests that *system*(*error*) is useful in finding relationships in the reference graph. These results suggest that the genome reference graph is very useful and gives a major contribution to the final result.

## Discussion

**Figure 7:**
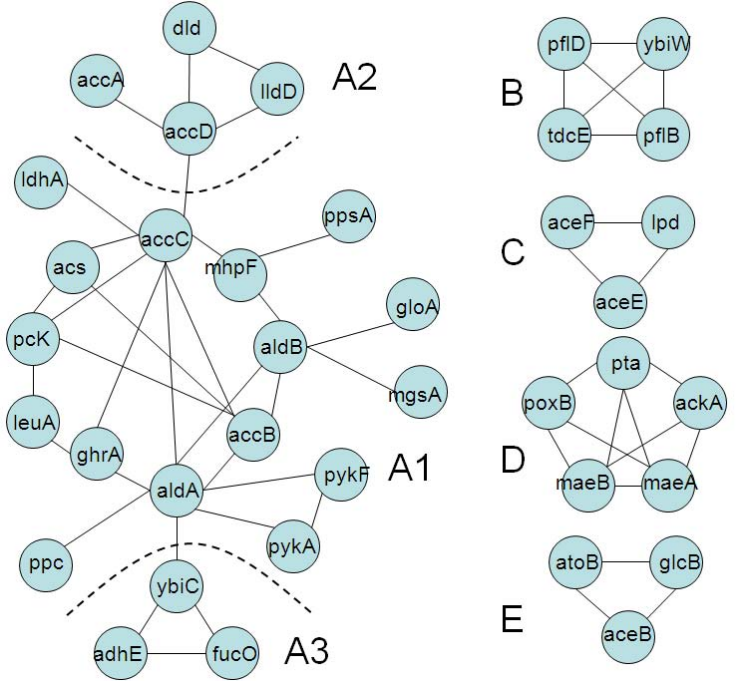
**Reconstructed gene connections of Eco00620**. Connected graph constructed by the recalled information predicted in our program. If a gene can be recalled by another gene in final top 10 ranks, then an edge is connected between the gene pair. 38 genes are recalled and connected as five submodules. The biggest module has 23 genes and can be further divided as three parts.

**Figure 8:**
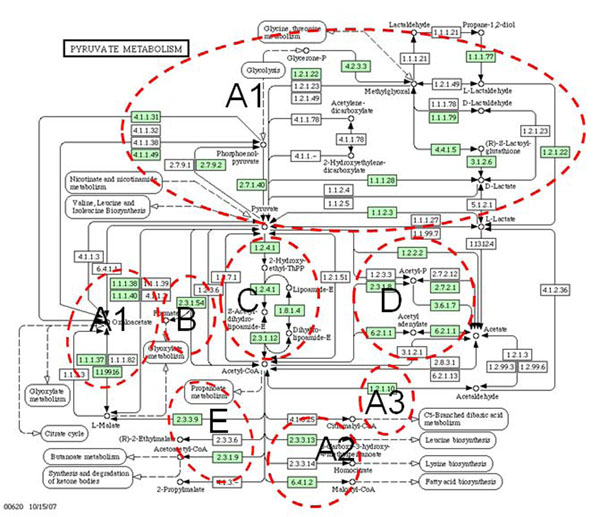
**Mapped structures on the pathway of Eco00620 in KEGG.** Five recalled modules can be well mapped on the described pathway Eco00620 in KEGG. Each module can be mapped with a biochemical process.

Our method provides new insights about finding missing genes and recruiting additional genes into partially predicted pathways in *E. coli*, through combining operon information and homology information across multiple genomes. Some wrongly predicted genes may indicate pathways might be quite functionally related (as being showed above, genes in eco00620 can recall many genes in eco00640), since pathways are defined quite arbitrary by biologists, this may remind us to think about the redefinition of some pathways. In some pathways, we noted that some genes form (connected) functional modules. We have systematically checked for this by connecting two genes with a link if one gene can be recalled by another gene among the top 10 predictions; and used all the genes in eco00620 to reconstruct a new graph with 5 connected components (Figure 7). The biggest component includes 23 genes and is the main functional module in pyruvate metabolism and can be further parted into three smaller sub-modules (A1, A2 and A3), and we found all sub-modules are indicating special biochemical processes (Figure 8). For example, part C includes three genes from the same operon and they are involved in the process to metabolize Pyruvate to Acetyl-CoA in eco00620. The structures observed in individual pathways and between pathways provide more insights about the hierarchical structure of pathways and consisted with earlier studies [24-26].

## Concluding remark

We present a method to find pathway genes at a genome level, which can be used to fill pathway holes or recruit new genes into existing pathways. The results show that our method can achieve higher prediction accuracy and is very robust. The main advantage of our method is that by introducing the reference graph, we get a natural way to integrate different types of information such as genomic structure information and sequence similarity information. More information could possibly be added in future studies. For example, we can use information like regulons [27] and gene fusion events [28] to provide a more general framework for integrating different information, which can be easily included into our current program. Besides finding new genes for pathways, our method can also be used for functional module inference, as some functional modules may be the union of existing pathways.

## Competing interests

The authors declare that they have no competing interests.

## Authors' contributions

Yong Chen produced the program and contributed towards planning and writing of the manuscript, particularly producing the Results section. Fenglou Mao and Guojun Li contributed in preparing data and some results analysis. Ying Xu provided guidance and planning for the project. All authors read and approved the final manuscript.

## Supplementary Material

Additional File 1 **the strain name and NCBI ID of 185 strains (genomes with plasmids)**. 185 strains (non-redundant genomes and plasmids) which have longest sequence in each genera are selected from 185 different (NCBI release of 9.2009). Click here for file

Additional File 2 **Names and gene number of 121 pathway genes.** 121 characterized pathways of *E. coli* K12 is downloaded from KEGG (released 9.2009). Click here for file

Additional File 3 **SP and SE value.** Calculated average SP and SE with constraints *system*(*error*) = 0.06, *α* = 380, *β* = 5, K is changed to 5, 10, 15, 20, 25, 30. Click here for file

Additional File 4 **The average PPV rate of *E.coli* pathways based on the 2nd level of KEGG orthology.** The pathways (|*G*(*P*) ≥ 5|) are calculated with *system*(*error*) = 0.06, *K* = 5, *α* = 380, *β* = 5, *γ* = 10. Click here for file

Additional File 5 **PPV values of individual pathway with |*G*(*P*)| ≥ 30.** The PPV values are calculated based on system(error) *system*(*error*) = 0.06, *K* = 5, *α* = 380, *β* = 5, *γ* = 10. Click here for file
