# Supplementary material for: Genome-wide discovery of missing genes in biological pathways of prokaryotes
Source: BMC Bioinformatics. 2011 Feb 15;12(Suppl 1):S1. doi: 10.1186/1471-2105-12-S1-S1 (PMC3044263; doi:10.1186/1471-2105-12-S1-S1)

Additional File3 – SP and SE value.

Calculated average SP and SE with constraints $system(error) = 0.06$, $\alpha = 380$, $\beta = 5$, K is changed to 5, 10, 15, 20, 25, 30.

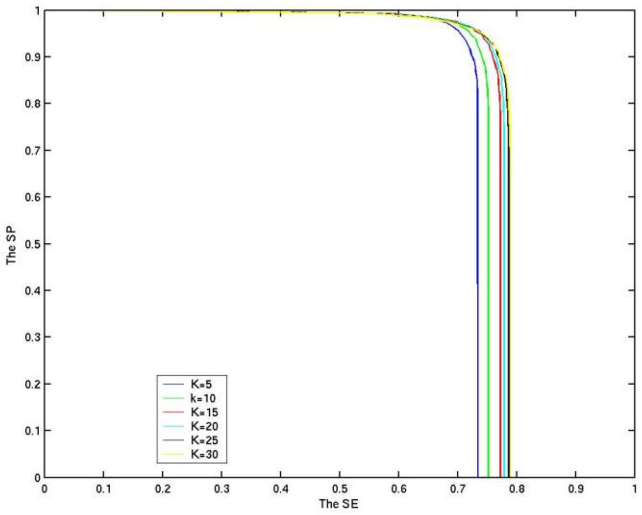

Supplement: Additional File 3 — SP and SE value. Calculated average SP and SE with constraints system(error) = 0.06, α = 380, β = 5, K is changed to 5, 10, 15, 20, 25, 30. [file 1471-2105-12-S1-S1-S3.pdf]
