# Supplementary material for: Genome-wide discovery of missing genes in biological pathways of prokaryotes
Source: BMC Bioinformatics. 2011 Feb 15;12(Suppl 1):S1. doi: 10.1186/1471-2105-12-S1-S1 (PMC3044263; doi:10.1186/1471-2105-12-S1-S1)

Additional File4 – The average PPV rate of *E.coli* pathways based on the 2nd level of KEGG orthology.

The pathways ($|G(P)| \geq 5$) are calculated with $system(error) = 0.06, K = 5, \alpha = 380, \beta = 5,$ $\gamma = 10$.

CM: Carbohydrate Metabolism, AAM: Amino Acid Metabolism, BPNP: Biosynthesis of Polyketides and Nonribosomal Peptides, BSM: Biosynthesis of Secondary Metabolism, CELLM: Cell motility, EM: Energy Metabolism, FSD: Folding Sorting and Degradation, GBM: Glycan Biosynthesis and Metabolism, LM: Lipid Metabolism, MT: Membrane Transport MCV: Metabolism of cofactors and Vitamins, MOAA: Metabolism of Other Amino Acids, NM: Nucleotide Metabolism RR: Replication and Repair, ST: Signal Transduction, TSC: Transcription, TSL: Translation, XBM: Xenobiotics Biodegradation and Metabolism.

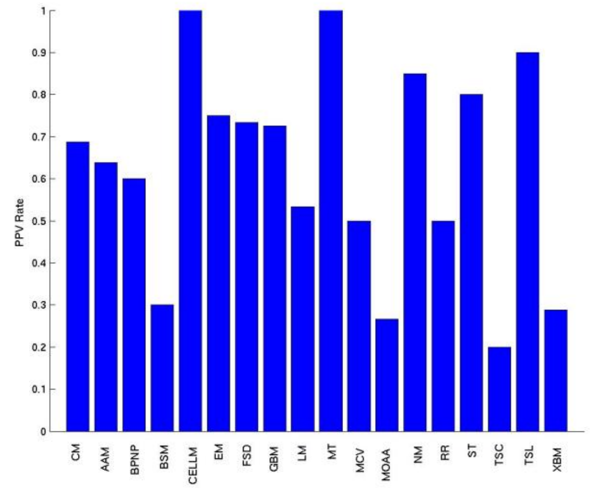

Supplement: Additional File 4 — The average PPV rate of E.coli pathways based on the 2nd level of KEGG orthology. The pathways (|G(P) ≥ 5|) are calculated with system(error) = 0.06, K = 5, α = 380, β = 5, γ = 10. [file 1471-2105-12-S1-S1-S4.pdf]
