# Supplementary material for: Genome-wide discovery of missing genes in biological pathways of prokaryotes
Source: BMC Bioinformatics. 2011 Feb 15;12(Suppl 1):S1. doi: 10.1186/1471-2105-12-S1-S1 (PMC3044263; doi:10.1186/1471-2105-12-S1-S1)

Additional File5 – PPV values of individual pathway with $|G(P)| \geq 30$.

The PPV values are calculated based on $system(error) = 0.06$, $K = 5$, $\alpha = 380$, $\beta = 5$, $\gamma = 10$.

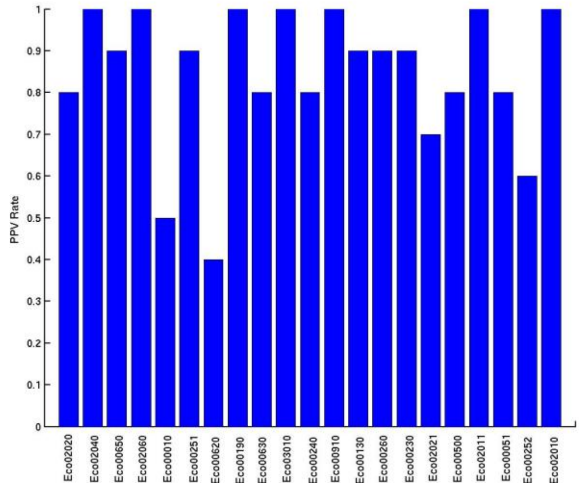

Supplement: Additional File 5 — PPV values of individual pathway with |G(P)| ≥ 30. The PPV values are calculated based on system(error) system(error) = 0.06, K = 5, α = 380, β = 5, γ = 10. [file 1471-2105-12-S1-S1-S5.pdf]
